# Genomic Variations in Drug Resistant *Mycobacterium tuberculosis* Strains Collected from Patients with Different Localization of Infection

**DOI:** 10.3390/antibiotics10010027

**Published:** 2020-12-31

**Authors:** Ekaterina Chernyaeva, Mikhail Rotkevich, Ksenia Krasheninnikova, Alla Lapidus, Dmitrii E. Polev, Natalia Solovieva, Viacheslav Zhuravlev, Piotr Yablonsky, Stephen J. O’Brien

**Affiliations:** 1 Medical Faculty, St. Petersburg State University, 199034 St. Petersburg, Russia; piotr_yablonskii@mail.ru; 2 Laboratory of Molecular Epidemiology and Evolutionary Genetics, St. Petersburg Pasteur Institute, 197101 St. Petersburg, Russia; 3 St. Petersburg Research Institute of Phthisiopulmonology, 197101 St. Petersburg, Russia; baclab@mail.ru (N.S.); jouravlev-slava@mail.ru (V.Z.); 4 Theodosius Dobzhansky Center for Genome Bioinformatics, St. Petersburg State University, 199034 St. Petersburg, Russia; rotke@yandex.ru (M.R.); krasheninnikova@gmail.com (K.K.); 5 Laboratory of Genomics Diversity, Center for Computer Technologies, ITMO, 197701 St. Petersburg, Russia; lgdchief@gmail.com; 6 Center for Algorithmic Biotechnology, St. Petersburg State University, 199034 St. Petersburg, Russia; a.lapidus@spbu.ru; 7 Resource Center «Biobank», Research Park of St. Petersburg State University, 199034 St. Petersburg, Russia; brantoza@gmail.com; 8 Guy Harvey Oceanographic Center Halmos, College of Arts and Sciences, Nova Southeastern University, Ft Lauderdale, FL 33004, USA

**Keywords:** *Mycobacterium tuberculosis*, drug resistance, pulmonary tuberculosis, extrapulmonary tuberculosis, whole-genome sequencing

## Abstract

*Mycobacterium tuberculosis* is a highly studied pathogen due to public health importance. Despite this, problems like early drug resistance, diagnostics and treatment success prediction are still not fully resolved. Here, we analyze the incidence of point mutations widely used for drug resistance detection in laboratory practice and conduct comparative analysis of whole-genome sequence (WGS) for clinical *M. tuberculosis* strains collected from patients with pulmonary tuberculosis (PTB) and extra-pulmonary tuberculosis (XPTB) localization. A total of 72 pulmonary and 73 extrapulmonary microbiologically characterized *M. tuberculosis* isolates were collected from patients from 2007 to 2014 in Russia. Genomic DNA was used for WGS and obtained data allowed identifying major mutations known to be associated with drug resistance to first-line and second-line antituberculous drugs. In some cases previously described mutations were not identified. Using genome-based phylogenetic analysis we identified *M. tuberculosis* substrains associated with distinctions in the occurrence in PTB vs. XPTB cases. Phylogenetic analyses did reveal *M. tuberculosis* genetic substrains associated with TB localization. XPTB was associated with Beijing sublineages Central Asia (Beijing CAO), Central Asia Clade A (Beijing A) and 4.8 groups, while PTB localization was associated with group LAM (4.3). Further, the XPTB strain in some cases showed elevated drug resistance patterns relative to PTB isolates. HIV was significantly associated with the development of XPTB in the Beijing B0/W148 group and among unclustered Beijing isolates.

## 1. Introduction

*Mycobacterium tuberculosis* is one of the most widespread and studied pathogens across the globe. According to the WHO estimation, Russia had about 79,000 new tuberculosis (TB) cases (case rate 54 per 100,000) and 10,500 TB deaths in 2018, with 28% of new TB cases displaying multiple drug resistance or resistance to rifampicin (MDR/RR-TB) [1]. The main clinical form of tuberculosis (TB), pulmonary tuberculosis (PTB) is considered to be the most epidemically dangerous localization of the disease. For many years the proportion of patients with XPTB has remained constant with a variation of <3% in Russia [2]. The relatively low rate of extra-pulmonary tuberculosis (XPTB) in Russia could be explained by the fact that TB of intrathoracic lymph nodes and tuberculous pleurisy are generally not counted in XPTB statistics. Delayed detection of XPTB leads to a high proportion of chronic TB forms and to a high level of disability among patients. The most common localization of XPTB is an osteoarticular localization, which is detected in 35.7% cases of all XPTB cases [2]. Pulmonary or extrapulmonary localization of the tuberculous process might be influenced by differences of the host, the immune status and genome features, as well as with the biological or genetic differences of the pathogen. 

In this study we compare the genomic landscape of clinical *M. tuberculosis* strains with various drug susceptibility profiles collected from patients with PTB and XPTB localization based on whole-genome sequencing data. XPTB *M. tuberculosis* WGS data were previously described by our team [3]. 

## 2. Results

### 2.1. M. tuberculosis Phylogenetic Analysis

**Figure 1:**
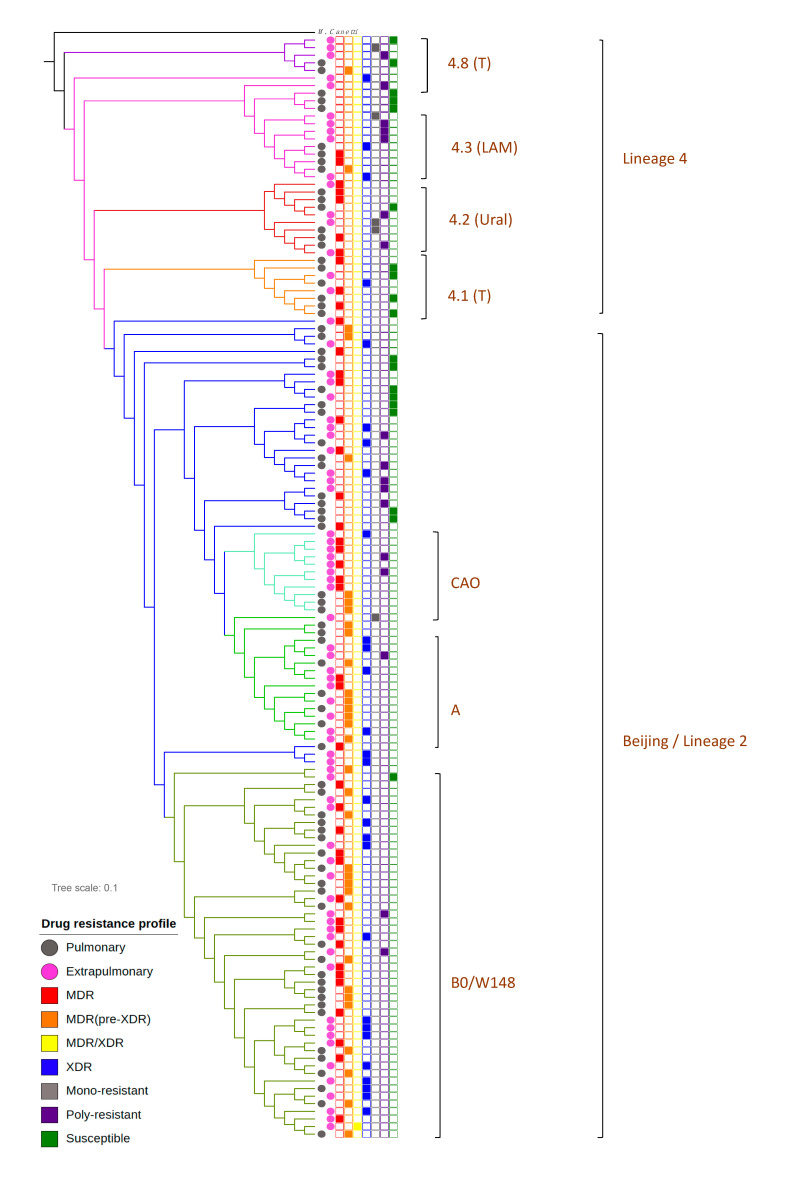
Phylogenetic analysis of *M. tuberculosis* isolates obtained from patients with pulmonary tuberculosis (PTB) and extra-pulmonary tuberculosis (XPTB).

Phylogenetic comparative analysis was performed to give a characterization of studied bacterial population structure. Maximum likelihood estimation based on WGS data allowed us to discriminate two large lineages among sequenced *M. tuberculosis* isolates–2 and 4 (Figure 1). Lineage 4 was represented by four monophyletic clusters which belonged to phylogenetic groups 4.1, 4.2, 4.3 and 4.8 based on PhyTB classification [4] and one isolate belonged to 4.4 phylogenetic groups. Major lineage 2 (Beijing) was represented by isolates belonged to the 2.2.1 group based on PhyTB classification. Phylogenetic analysis based on 8673 SNVs allowed us to distinguish several subclusters in lineage 2. Analysis of DNA markers such as regions of difference RD105, RD207, RD181 and mutT2 and mutT4 genes [5] allowed us to classify ancient and modern sublineages within the Beijing genotype (Figure 1). The largest subgroup within the Beijing clade was a group of strains that belonged to B0/W148 genetic lineage (Figure 1). The B0/W148 genetic cluster is characterized by a specific IS*6110* insertion in genome position 2,982,598 in the *Rv2664*-*Rv2665* intergenic region [6]. Besides the Beijing B0/W148 sublineage we identified clusters belonging to Beijing sublineages Central Asia (Beijing CAO) and Central Asia Clade A (Beijing A) based on single nucleotide variations (SNV) classification published by Shitikov et al. [7].

Phylogenetic groups were identified using a maximum-likelihood approach. Isolates obtained from patients with PTB are marked using the pink square symbol and from XPTB with the black square symbol. Beijing genetic group (lineage 2), colored with the blue symbol, could be discriminated on three subclusters (B0/W148, Clades A and CAO marked with different shades of green) and a group of unclustered strains. Lineage 4 is represented by Ural (red), LAM (pink color) and T genetic families, whereas one isolate belongs to genetic group 4.4, spoligotype family S. 

Detailed information on *M. tuberculosis* genetic lineages, microbiological and clinical data are available in Supplementary Table S1.

The Beijing genetic group was predominant among both pulmonary and extrapulmonary isolates (Table 1). However, Beijing isolates were significantly more frequent among XPTB patients (82.19%, *n* = 60) than among PTB patients (66.67%, *n =* 48, *p* = 0.03721). A large proportion of *M. tuberculosis* Beijing isolates (45.37%, *n* = 59) belonged to the B0/W148 subcluster. The Beijing B0/W148 group was equally represented in PTB vs. XPTB groups of bacterial isolates, while the frequency of Beijing sublineages CAO and Clade A was much higher among XPTB isolates. Beijing CAO strains were identified among 4.19% (*n* = 3) PTB and 10.96% (*n* = 8) XPTB cases. Beijing Clade A was detected among 4.19% (*n* = 3) PTB and 19.18% (*n* = 14) XPTB cases. Comparative analysis of XPTB cases caused by bacterial isolates from 4.8 and 4.3 genetic groups revealed significant differences in these clusters. *M. tuberculosis* isolates from 4.8 subcluster caused XPTB more often than isolates from the 4.3 subcluster (*p* = 0.02171). *M. tuberculosis* isolates from phylogenetic subcluster 4.3 were more often detected among PTB cases. The Beijing Clade A was associated with XPTB, while 4.3 sublineage was associated with PTB cases (FET Beijing A vs. 4.3: *p* = 0.0005944). A similar positive association was observed for XPTB with *M. tuberculosis* sublineages Beijing CAO and 4.3, while subclade 4.3 was strongly associated with PTB (Beijing CAO vs. 4.3: *p* = 0.01109). 

Results of phylogenetic analysis was used for screening for synapomorphic SNVs and Indels. Statistical analysis did not identify specific genomic signatures (SNVs or InDels) associated with TB tissue localization across different *M. tuberculosis* phylogenetic groups (Supplementary Figure S1). 

### 2.2. Drug Resistance of PTB and XPTB Strains

*M. tuberculosis* drug susceptibility testing allowed the recognition of five clinical groups of isolates: (a) susceptible; (b) monoresistant, i.e., resistant to one drug; (c) poly-resistant—resistant to more than one drug, but not MDR; (d) multidrug-resistant (MDR) and (e) extensively drug-resistant (XDR), according to the WHO definition [8,9] (Table 2). The prevalence of the XDR phenotype was almost 5-fold higher in PTB than was observed in XPTB cases (33% XDR in PTB vs. 6.8% XDR in XPTB; *p* = 6.133 × 10^−5^; Table 2). 

The majority of bacterial isolates in the Beijing cluster were resistant to at least one drug. Over 80% of *M. tuberculosis* isolates in Beijing cluster had MDR (*n* = 62) and XDR (*n* = 25). Only nine out of 108 isolates were susceptible to all drugs (Table 3). Statistical analysis showed an association of the Beijing cluster with drug resistance (*p* = 0.008542) and especially with M/XDR (*p* = 3.007 × 10^−5^) compared to nonBeijing clades. 

### 2.3. Mutations Associated with Drug Resistance

WGS data were screened for previously published mutations associated with resistance to TB drugs [10]: streptomycin (SM), isoniazid (INH), rifampicin (RIF), ofloxacin (OFL), pyrazinamide (PZA), ethambutol (EMB), ethionamide (ETH), kanamycin (KM), amikacin (AM), cycloserine (CS), capreomycin (CM) and para-aminosalicylic acid (PAS) (Supplementary Tables S2 and S3). 

INH-resistance has been associated with mutation S315T in the *katG* gene, and 95.87% of INH-resistant strains in this study carried mutations in this region. RIF-resistance was associated with mutations in *rpoB* 81-bp core region and 96.15% of resistant isolates carried mutations in this genome region. Over 98% of SM-resistant isolates had mutations in *rpsL*, *rrs* and *gid* genes. Mutations in promoter region of *eis* gene were identified in 18 KM-resistant and 13 KM-susceptible isolates. It was previously shown that mutations in the regulatory region of *whiB7* gene can indirectly influence KM-resistance. However they are more likely are associated with SM-resistance [11]. We identified only two KM-resistant and three susceptible isolates with mutations in the region upstream *whiB7*, and all of these mutants were resistant to SM. 

The majority of OFL-resistant isolates (90.48%) carried mutations in *gyrA* or *gyrB* quinolone resistance-determining regions. Mutations in the *embB* gene between codones 296 and 497 [12] were detected in 63 resistant and 27 susceptible to EMB isolates. Mutations in the *embC*-*embA* intergenic region (8–16 nucleotides upstream *embA* gene) were detected in 16 EMB-resistant and seven EMB-susceptible isolates. Point mutations were detected in *pncA* and *rpsA* genes in 31 PZA-resistant isolates (67.39%). 

A list of SNVs that might be associated with PZA-resistance in *pncA* gene published by Yadon A. et al., 2017 [13] were subjects for discovery in the *M. tuberculosis* WGS data. We identified 41 mutations leading to amino acid substitutions in 62 *M. tuberculosis* isolates. Twenty-nine PZA-resistant isolates (63.04%) carried mutations in *pncA* gene. However three of these mutations were previously identified as associated with PZA-susceptibility and six mutations were not related to susceptibility or resistance. Nine PZA-susceptible isolates had SNVs in *pncA* gene; six of these mutations were associated with *in vivo* or/and *in vitro* resistance to PZA and three did not have association with PZA-susceptibility or resistance, according to Yadon et al. [13]. Among *M. tuberculosis* isolates with unknown PZA-resistance status (*n* = 24), 14 mutations associated with PZA resistance in 16 *M. tuberculosis* isolates were detected, one mutation in association with PZA-susceptibility and six mutations in eight genomes with unknown association with resistance or susceptibility to PZA (Supplementary Table S3). 

ETH-resistant isolates are known to have mutations located in *ethA/R* locus, *inhA* gene and its promoter [10]. Forty-eight sequenced isolates were known to be resistant to ETH. In our dataset, ETH-resistant isolates did not have mutations in the *ethA* gene. Mutations in the *fabG–inhA* operon (-15 and -34 nt) were detected in nine ETH-resistant and five ETH-susceptible isolates. Mutations in the *inhA* gene were relatively rare and were detected in two resistant and four susceptible isolates. Mutations in the *ethA* gene were identified in eight ETH-resistant, 25 ETH-susceptible and three isolates with unknown ETH susceptibility data. A mutation in the *ethR* gene (A70T) was detected only in one ETH-resistant isolate, while mutations in the *ethA-ethR* intergenic region (7 bp upstream *ethA* or 69 bp upstream *ethR* start-codons) was detected in nine isolates and only three of them were ETH-resistant.

We compared mutations, associated with *M. tuberculosis* drug resistance, detected in our study with a list of mutations that are widely used in molecular-genetic tests for *M. tuberculosis* identification and drug resistance prediction: HAIN GenoType MTBDRplus and GenoType MTBDRsl v1.0 and v2.0. The majority of mutations associated with INH-resistance that were detected in our study could be detected by the HAIN assay. However a mutation in the -34 position upstream of the *fabG-inhA* operon is not included in the HAIN catalogue and may be due to a relatively rare occurrence (two isolates in our dataset). Detection of mutations associated with RIF-resistance using the HAIN assay would also allow us to identify most of the RIF-resistant isolates in our dataset. However, a Q432K mutation in the *rpoB* gene was detected in one RIF-resistant strain and might be related with RIF-resistance. Mutations associated with EMB-resistance detected by the HAIN diagnostic assay are represented by M306V and M306I substitutions only. In our study M306V was the most frequent mutation (*n* = 20), M306I substitutions were identified in five genomes, while 38 *M. tuberculosis* isolates that had mutations in the *embB* region between codons 296 and 497 could not be detected by the HAIN system. Mutations in the *embA* promoter region that are not represented in HAIN test-system were detected in 14 EMB-resistant isolates. It should be noted that mutations in the *embB* gene and *embA* promoter associated with EMB-resistance are often detected among EMB-susceptible isolates. For instance, mutation M306V in the *embB* gene was detected in 20 EMB-resistant and nine EMB-susceptible isolates. Mutations associated with resistance to aminoglycosides and peptide antibiotics are represented in the HAIN test by three mutations in the *rrs* gene, associated with KM/AM/CM/viomycin resistance, and *eis* promoter mutations associated with KM resistance. We did not find mutations in *rrs* positions 1401, 1402 and 1484, but identified a mutation in the 1490 position in one CM-resistant isolate. Three out of four SNVs identified in the *eis* promoter could be detected by the HAIN assay (10, 12 and 14 bp upstream *eis* start codon). However, in our study these mutations were detected in 18 KM-resistant and 12 KM-susceptible isolates. The GenoType MTBDRsl assay allows detection of mutations in *gyrA* 90, 91 and 94 codons and in *gyrB* 538 and 540 codons associated with OFL-resistance. In our dataset most of the OFL-resistance mutations were detected in the *gyrA* gene and corresponded to the variety of mutations presented in the HAIN system, though mutations in *gyrB* gene that were detected in several OFL-resistant strains were not covered by the HAIN line-probe assay. 

### 2.4. TB/HIV Coinfection

Bacterial isolates were collected from 120 patients negative for human immunodeficiency virus (HIV) and 25 HIV-infected patients. We observed a higher rate of generalized TB among HIV-infected individuals (Table 4) (*p* = 6.579 × 10^−5^). XPTB was significantly more frequent among HIV-positive patients compared to patients with PTB (Table 4; *p* = 0.000998). Thus, HIV-infection increases the probability of TB generalization or development of active XPTB. XPTB development was significantly higher among patients infected with HIV carrying the Beijing-unclustered strain of *M. tuberculosis* (*p* = 0.068) (Table 5). A similar XPTB excess was apparent among patients carrying the Beijing B0/W148 strain (*p* = 0.0013) (Table 5). Other genetic groups with HIV coinfection did not make a significant impact on pulmonary or extrapulmonary TB development (Table 5). 

## 3. Discussion

The study aimed to compare the genomic landscapes of *M. tuberculosis* isolates obtained from patients with different clinical features of the disease: pulmonary and extrapulmonary TB. Our previous analysis of *M. tuberculosis* isolates from patients with tuberculous spondylitis showed that this group of bacterial strains is characterized by low genetic diversity and high prevalence of Beijing isolates (82% of strains belonged to the Beijing clade) [3]. Prevalence of the Beijing genetic group among extrapulmonary *M. tuberculosis* isolates compared to pulmonary ones was also reported in a recent study, which identified Beijing in 75% of the extrapulmonary *M. tuberculosis* cases [14]. Our current study showed that extrapulmonary *M. tuberculosis* isolates belonged to the Beijing genetic group (82.2%) more often than pulmonary (66.7%). Despite this there was a higher prevalence of Beijing strains among extrapulmonary *M. tuberculosis* samples (82.19% vs. 66.67%); in our study pulmonary strains were frequently associated with XDR (*p* = 6.133 × 10^−5^). It was previously shown that *M. tuberculosis* strains from the Beijing genetic group were highly associated with M/XDR [15,16]. Our results confirm this statement: the frequency of drug resistance among *M. tuberculosis* Beijing isolates was higher compared to other genetic groups in both pulmonary and extrapulmonary strains (Table 1). The *M. tuberculosis* genetic lineage 4 identified in our study was also previously detected in Russia: Ural family (4.2) identified in many regions in Eurasia [17], and heterogeneous sublineage 4.1 and sublineage 4.3 were observed in many countries across the world [17]. Like 4.2, sublineage 4.4 occurred in high proportions among isolates from particular countries in Asia and Africa but were largely absent from the Americas. Sublineage 4.8 was identified as a part of the 4.10 genetic group. The latter was previously mentioned as one of the most widely spread subclades of the lineage 4 [18].

It was shown that Beijing A and CAO isolates were significantly more often identified in extrapulmonary TB cases, while isolates from principal genetic lineage 4.3 were mostly identified in patients with pulmonary TB (Table 1). Association of specific genetic clades and subclades of *M. tuberculosis* strains with TB diagnosis, shown in our dataset, is an interesting fact which suggests some specific molecular signatures in different *M. tuberculosis* genetic groups that can make an impact on active TB disease localization, at least in the Russian population of TB patients. 

Analysis of mutations associated with bacterial susceptibility to first and second line TB drugs allowed us to identify that relatively high proportion of INH-, RIF-, SM- and OFL-resistant isolates had standard SNVs predictive of drug-resistance. However, some SNVs associated with resistance to TB drugs could not be unequivocally interpreted. For example, the proportion of EMB-resistant isolates with mutations in the *embB* gene between codones 296 and 497 and the *embC-embA* intergenic region were identified in 94.29% of EMB-resistant isolates. However, over 40% of EMB-susceptible isolates carried the same mutations. A similar situation was revealed with mutations associated with KM-resistance in *eis* and *whiB7* promoter regions—42.5% of KM-resistant and 22.4% of KM-susceptible isolates carried mutations in these regions (ST3). This observation might be related to the fact that mutations can be responsible for the development of high and low levels of drug resistance, while in our study we did not have data on minimum inhibitory concentrations. *M. tuberculosis* isolates from our dataset were checked for mutations in *pncA* and *rpsA* genes that were known to be involved in development of resistance to PZA [19]. Only four PZA-resistant isolates carried mutations in the *rpsA* gene, but the majority of PZA-resistant isolates had mutations in the *pncA* gene mentioned in a previously published catalog of mutations associated with susceptibility to PZA [13]. Despite this, most of the obtained data were consistent with previously published data. In PZA-resistant strains we detected few mutations that were mentioned as associated with susceptibility to PZA, and there were several PZA-susceptible isolates that carried mutations associated with PZA-resistance according to Yadon et al. data. This fact may indicate either that these mutations are not involved into the development of PZA resistance, or their influence on resistance is associated with higher doses of the drug that were analyzed in our research. Detection of mutations associated with drug resistance among susceptible isolates may indicate the presence of a low number of drug resistant clones in the *M. tuberculosis* population and might be a signal for correction of TB therapy in case it was developed based on phenotypic data. 

Comparative analysis of *M. tuberculosis* isolates obtained from HIV-positive and HIV-negative patients would suggest that HIV coinfection increases the probability of TB generalization, or development of XPTB. Previous epidemiological studies showed similar results in that a number of studies identified a relationship between HIV and the development of XPTB [20,21]. Results of recent research showed that HIV infection is one of risk factors for XPTB in the US [22]. The majority of *M. tuberculosis* strains included in our study (both pulmonary and extrapulmonary) belonged to the Beijing genetic group widely spread in Russia. Comparative analysis of XPTB and PTB cases in HIV-positive and HIV-negative patients infected with *M. tuberculosis* strains from various genetic groups allowed us to obtain different results. For example, HIV correlated with XPTB in patients infected with Beijing B0/W148 and unclustered Beijing strains. However, HIV coinfection was not associated with XPTB in patients infected with bacterial strains related to genetic clades Beijing CAO, A and 4.8 (associated with XPTB), and strains from 4.3 clade (associated with PTB). Thus, according to our data, it may be possible to assess the risk of XPTB or generalized TB depending on the pathogen strain and the presence of HIV coinfection. A limitation of our study was a relatively low number of *M. tuberculosis* isolates obtained from HIV-infected individuals. We suggest that further analysis of TB/HIV coinfection with attention on *M. tuberculosis* phylogeny could help better understand processes leading to active XPTB and generalized TB development. 

XPTB development is a multifactorial process that can depend on many factors, including the biology of the pathogen and the host. Understanding these factors can help in assessing the risks of developing complicated forms of TB, optimizing diagnosis and treatment. Our research allowed us to make a snapshot of genomic markers identified in *M. tuberculosis* strains obtained from patients with PTB and XPTB in Russia. Further comprehensive analysis of bacterial and human biological signatures might allow for better understanding consistent pattern of XPTB development.

## 4. Materials and Methods 

A total of 72 PTB and 73 XPTB isolates were collected between 2007 to 2014 from 40 different regions of the Russia (Figure 1, Supplementary Table S1). Isolates were selected randomly from the *M. tuberculosis* strains collection of St. Petersburg Research Institute of Phthisiopulmonology. HIV status was known for each patient involved in the study, since all TB patients in Russia are tested for HIV. Eighteen isolates received from XPTB localization were collected from patient with generalized TB (disseminated form of TB). The majority of studies isolates (*n* = 120) were obtained from HIV-negative patients and 25 from HIV-infected patients. 

Bacterial isolates were cultured from pulmonary and extrapulmonary clinical material. The susceptibility of *M. tuberculosis* cultures to isoniazid (INH), rifampicin (RIF), streptomycin (SM), ethambutol (EMB), pyrazinamide (PZA), ethionamide (ETH), ofloxacin (OFL), kanamycin (KM), amikacin (AM), cycloserine (CS), capreomycin (CM) and para-aminosalicylic acid (PAS) was detected using WHO recommendations [23] and Russian regulatory documents. 

Phenotypic drug susceptibility testing was performed using solid Lowenstein-Jensen (LJ) and liquid BACTEC MIGIT 960 (MIGIT) media. A drug resistance test was performed depending on which medium the growth of the clinical strain was obtained. Susceptibility of *M. tuberculosis* isolates was studied according to manufacturer’s recommendations on MIGIT media and using absolute concentration method on LJ media. The following concentrations (μg/mL) of each anti-TB drugs were used:

INH—1.0 (LJ), 0.1 (MIGIT)
RIF—40.0(LJ), 1.0 (MIGIT)
SM—10.0 (LJ), 1.0 (MIGIT)
EMB—2.0 (LJ), 5.0 (MIGIT)
PZA—100.0 (MIGIT), LJ was not used
ETH—40.0 (LJ), 5.0 (MIGIT)
OFL—2.0 (LJ), 2.0 (MIGIT)
KM—30.0 (LJ), 2.5 (MIGIT)
AM—1.0 (MIGIT), LJ was not used
CS—30.0 (LJ), MIGIT was not used
CAP—30.0 (LJ), 2.5 (MIGIT)
PAS—1.0 (LJ), MIGIT was not used

*M. tuberculosis* genomic DNA was extracted using lysis with proteinase K and cetyl trimethylammonium bromide (CTAB) with further phenol/chloroform extraction and alcohol precipitation [24]. Bacterial DNA was subjected to WGS using MiSeq platform (Illumina). *M. tuberculosis* WGS data were deposited in the NCBI SRA (PRJNA352769). 

The quality of *M. tuberculosis* sequence reads was evaluated using FastQC (v. 0.11.7) [25]. Sequence reads were processed using Trimmomatic [26] with the ILLUMINACLIP adapter-clipping setting 2:30:10 LEADING:3 TRAILING:3 SLIDINGWINDOW:10:20. We aligned sequenced reads to the reference genome of *M. tuberculosis* H37R (NC_000962.3) and called variants (single-nucleotide polymorphisms [SNPs] and short insertions/deletions) by using bioinformatics software: bowtie2 (v. 2.3) [27] with a very-sensitive mode; SAMtools [28] and VCFtools [29] with default parameters; and FreeBayes [30]. This tool was preferred due to its haplotype-based approach, which is desired for haploid genome analysis and explicit output format. Except for pointing out the haploidy (-p 1) and excluding the repetitive regions, the other parameters were set to default. Variant Call Format (VCF) files were used for comprehensive genetic analysis. We used mutations that had q-scores ≥20 for comprehensive analysis. We used concatenated SNPs for phylogenetic analysis by using the GTRCAT (general time-reversible model with rate heterogeneity accommodated by using discrete rate categories) maximum-likelihood algorithm from the RAxML software package [31] to calculate an approximation model and 100 bootstrap replications. To avoid misalignments, we annotated SNPs in repetitive genome regions and in genes encoding proteins that contain proline-glutamate or proline-proline-glutamate motifs and filtered them from analysis. We used PhyTB [4] and SpoTyping tools [32] for phylogenetic classification of *M. tuberculosis* genomes. R commander package for R [33] and Python scripts were used for statistical analysis using Fisher exact tests (FET) for significant statistical association.

## Figures and Tables

**Table 1:** Genetic clades, identified among *M. tuberculosis* isolates from different localizations.

	PTB		XPTB		Total	
Genotype	N	%	N	%	N	%
Beijing:	48	66.67	60	82.19	108	74.48
Beijing unclustered	16	22.22	15	20.55	31	21.38
Beijing B0/W148	26	36.11	23	31.51	49	33.79
Beijing Clade A	3	4.17	14	19.18	17	11.72
Beijing CAO	3	4.17	8	10.96	11	7.59
4.1	5	6.94	3	4.11	8	5.52
4.2	6	8.33	4	5.48	10	6.9
4.3	11	15.27	2	2.74	13	8.97
4.4	1	1.39	0	0	1	0.69
4.8	1	1.39	4	5.48	5	3.45

**Table 2:** Characterization of drug susceptibility of *M. tuberculosis* from different localizations, n (%).

Diagnosis	Susceptible	Mono-Resistant	Poly-Resistant	MDR	XDR	Total
Pulmonary	12 (16.44)	3 (4.11)	9 (12.33)	44 (60.27)	5 (6.85)	73
Extrapulmonary	7 (9.72)	2 (2.78)	9 (12.5)	30 (41.67)	24 (33.33)	72
Total number	19 (13.10)	5 (3.45)	18 (12.41)	74 (51.03)	29 (20.00)	145

**Table 3:** Characterization of drug susceptibility in different *M. tuberculosis* genetic clades (n).

Genotype	Susceptible	Mono- Resistant	Poly- Resistant	MDR	XDR	Total Number
Beijing:	9	1	11	62	25	108
Beijing unclustered	8	0	5	12	6	31
Beijing B0/W148	1	0	3	31	14	49
Beijing A	0	1	1	11	4	17
Beijing CAO	0	0	2	8	1	11
4.1	4	0	0	3	1	8
4.2	1	2	2	5	0	10
4.3	3	1	4	3	2	13
4.4	0	0	0	0	1	1
4.8	2	1	1	1	0	5

**Table 4:** Number of TB/HIV coinfection cases.

Diagnosis	HIV+	HIV−	N
PTB	3	69	72
XPTB	14	41	55
Generalized TB	8	10	18

**Table 5:** Number of *M. tuberculosis* isolates obtained from patients with different TB localization and HIV-status.

Geneinc Clade	HIV Coinfection	Diagnosis	
		PTB	XPTB
4.8	+	0	1
	−	1	3
4.3	+	1	1
	−	10	1
4.2	+	1	0
	−	5	4
4.1	+	0	0
	−	5	3
4.4	+	0	0
	−	1	0
Beijing unclustered	+	0	6
	−	16	9
Beijing B0/W148	+	1	10
	−	25	13
Beijing CAO	+	0	1
	−	4	7
Beijing A	+	0	3
	−	2	11
Total		72	73

## Data Availability

WGS data represented in this study are openly available in the NCBI SRA (PRJNA352769).

## Supplementary Materials

The following are available online at https://www.mdpi.com/2079-6382/10/1/27/s1, Table S1: Phenotypic and clinical information on 145 *M. tuberculosis* isolates included into the study, Table S2: Mutations associated with drug resistance detected in individual *M. tuberculosis* genomes, Table S3: Counts of drug resistance mutations discovered among *M. tuberculosis* isolates with different susceptibility, Figure S1: Genome-wide Association Study Manhattan plot.
